# Dodecafluoropentane Emulsion Elicits Cardiac Protection Against Myocardial Infarction Through an ATP-Sensitive K^+^ Channel Dependent Mechanism

**DOI:** 10.1007/s10557-014-6557-2

**Published:** 2014-10-16

**Authors:** Joshua Strom, Trevor Swyers, David Wilson, Evan Unger, Qin M. Chen, Douglas F. Larson

**Affiliations:** 1 Department of Pharmacology; College of Medicine, University of Arizona, 1501 N. Campbell Ave, Tucson, AZ 85724 USA; 2 Department of Radiology; College of Medicine, University of Arizona, 1501 N. Campbell Ave, Tucson, AZ 85724 USA; 3 Sarver Heart Center; College of Medicine, The University of Arizona, 1501 N. Campbell Ave, Tucson, AZ 85724 USA

**Keywords:** Myocardial infarction, Perfluorocarbons, Dodecafluoropentane

## Abstract

**Purpose:**

Dodecafluoropentane emulsion (DDFPe) is a perfluorocarbon with high oxygen dissolving, transport, and delivery capacity that may offer the potential to limit ischemic injury prior to clinical reperfusion. Here we investigated the cardiac protective potential of DDFPe in a mouse model of myocardial infarction.

**Methods:**

Myocardial infarction was initiated by permanent ligation of the left anterior descending (LAD) coronary artery. Mice were administered vehicle or 5-hydroxydecanoate (5-HD) intravenously 10 min before LAD occlusion followed by a single intravenous administration of vehicle or DDFPe immediately after occlusion. Heart tissue and serum samples were collected 24 after LAD occlusion for measurement of infarct size and cardiac troponin I (cTnI) levels, respectively.

**Results:**

DDFPe treatment reduced infarct size by approximately 72 % (36.9 ± 4.2 % for vehicle vs 10.4 ± 2.3 % for DDFPe; *p* < 0.01; *n* = 6–8) at 24 h. Serum cTnI levels were similarly reduced by DDFPe (35.0 ± 4.6 ng/ml for vehicle vs 15.8 ± 1.6 ng/ml for DDFPe; *p* < 0.01; *n* = 6–8). Pretreatment with 5-HD, a mitochondrial ATP-sensitive potassium channel (mitoK_ATP_) inhibitor, blocked the reduction in infarct size (29.2 ± 4.4 % for 5-HD vs 35.4 ± 7.4 % for 5-HD+DDFPe; *p* = 0.48; *n* = 6–8) and serum cTnI levels (27.4 ± 5.1 ng/ml for 5-HD vs 34.6 ± 5.3 ng/ml for 5-HD+DDFPe; *p* = 0.86; *n* = 6–8) by DDFPe.

**Conclusion:**

Our data indicate a cardiac protective role of DDFPe that persists beyond its retention time in the body and is dependent on mitoK_ATP_, an important mediator of ischemic preconditioning induced cardiac protection.

## Introduction

Myocardial infarction as a result of coronary heart disease is a serious health concern, accounting for more deaths each year than any other individual disease [1]. Myocardial infarction occurs due to occlusion of one or more arteries, typically a coronary or branching artery, supplying the myocardium. Arterial occlusion results in ischemia of the downstream tissue, which cause myocardial damage and necrosis. Since the heart is a terminally differentiated tissue with limited regenerative potential, any loss of cardiac myocytes and contractile machinery can adversely impact cardiac function. It is therefore imperative that myocardial damage is limited, and the most effective therapy is the reperfusion of the ischemic tissue. Myocardial damage begins within 20 to 30 min of the ischemic period, with irreversible myocyte necrosis occurring over the course of several hours. The best clinical outcomes are observed when reperfusion is initiated within 2 h after the onset of symptoms [2, 3]. Percutaneous coronary intervention (PCI) is a mainstay for reperfusion therapy. One issue with PCI is the time between first medical contact and the start of the procedure, or door-to-balloon time. Reports indicate that the average door-to-balloon time for patients arriving at a facility capable of PCI is 60 min. In cases where inter-hospital transfer is required for PCI only 33 % have a door-to-balloon time of less than 90 min and only 66 % have a door-to-balloon time of less than 120 min [4]. Furthermore, the mean symptom duration time of MI before first medical contact is 2 h. As a result most patients receive intervention well outside of the optimal 2 h window, highlighting the need for therapeutic options that extend the timeframe for optimal reperfusion.

Perfluorocarbon (PFC) based oxygen carriers represent an intriguing option for treating ischemic conditions. PFCs are highly inert compounds with the ability to dissolve, transport, and deliver significant amounts of oxygen and other gases [5, 6]. PFC emulsions offer particular promise for oxygen delivery. Important for application to the treatment of ischemic tissue is the small particle size (~200 nm) of PFC emulsions that can be produced. The small size of these PFC emulsions may allow passage through many vascular occlusions that prevent the movement of larger red blood cells. A comparison of three PFC emulsions identified dodecafluoropentane emulsion (DDFPe) as having greater oxygen delivery capacity [7]. Among other PFCs, this increased oxygen capacity is unique to DDFPe, which undergoes gaseous expansion that increases intramolecule pockets for dissolving respiratory gasses at physiological temperatures.

The utility of DDFPe as an oxygen delivery system has been demonstrated in vivo. In addition, the therapeutic potential of DDFPe has been applied to supplement oxygen delivery and maintain tissue viability in severely anemic rats and to reduce infarct size in a rabbit model of ischemic stroke [8–10]. DDFPe represents a potential avenue to extend the timeframe of reperfusion therapy and reduce infarct size following a myocardial infarction through enhanced oxygen delivery prior to revascularization. Here we used a mouse model of myocardial infarction caused by permanent occlusion of the left anterior descending (LAD) coronary artery to investigate the potential of DDFPe as a cardiac protective agent in the absence of reperfusion.

## Materials and Methods

### Chemicals and Drugs

2,2,2-tribromoethanol (avertin) was diluted with 2-methyl-2-butanol and phosphate buffered saline (PBS) and was given intraperitoneally at a dose of 400 mg/kg. Avertin, 5-hydroxydecanoate (5-HD), and 2-methyl-2-butanol were purchased from Sigma-Aldrich (St. Louis, MO). 5-HD was diluted with PBS for a final concentration of 5 mg/ml. 5-HD was injected 5 min prior to LAD ligation at a dose of 10 mg/kg. 2 % (weight/volume) Dodecafluoropentane emulsion (DDFPe) was given at a dose of 0.6 ml/kg. DDFPe was diluted with PBS to a total volume of 200 μl prior to injection into the left jugular vein. DDFPe was a generous gift from NuvOx Pharma (Tucson, AZ). Buprenorphine slow release was used for intraoperative and postoperative pain control at the dose of 1 mg/kg given subcutaneously given immediately before the operation. Buprenorphine slow release was purchased through the University of Arizona’s animal care department.

### Surgical Procedure

Animal experiments were performed according to the protocol approved by the University of Arizona Institutional Animal Care and Use Committee. Studies conformed to the ‘Guide for the Care and Use of Laboratory Animals’ published by the US National Institutes of Health (NIH Publication No. 85-23, revised 1996). Male C57BL/6 J mice, age 8–12 weeks, were randomized into four groups: vehicle, DDFPe, 5-HD + vehicle, and 5-HD + DDFPe. Mice were administered saline (vehicle and DDFPe groups) or 5-HD (5-HD + vehicle and 5-HD + DDFPe groups) intravenously (IV) via the left jugular vein 5 min prior to occlusion of the left anterior descending coronary artery (LAD). The heart was visualized by a left thoracotomy and 8-0 suture was used to ligate the left anterior descending coronary artery. Immediately after LAD ligation, vehicle (vehicle and 5-HD + vehicle groups) or DDFPe (DDFPe and 5-HD + DDFPe groups) was administered IV via the left jugular vein. The chest cavity was closed and the animal was allowed to recover for 24 h. Mice were sacrificed 24 h after LAD ligation for collection of blood and infarct quantification.

### Infarct Quantification

Trypan blue dye (2 %) was perfused through the heart via the descending aorta to stain the non-ischemic area. The hearts were then excised and cut in 1 mm cross sections followed by staining in 1.5 % triphenyl tetrazolium chloride (TTC) for 30 min at 37 °C. Following TTC staining the slices were fixed in 10 % formalin and photographed. Left ventricle area, area at risk, and infarct size were quantified using ImageJ (NIH) planimetry. Area at risk was determined from the area not stained by trypan blue. Infarct was represented by the area at risk not stained red by TTC. Heart collection and infarct quantification was performed by an individual blinded from the surgical procedure and drug administrations.

### Western Blot Analysis

Blood plasma protein concentrations were measured using the Bradford method (Bio-Rad). Equal protein amount of each sample were diluted in laemmli sample buffer (25 % v/v glycerol, 2 % w/v SDS, 0.01 % w/v bromophenol blue, 350 mM dithiothreitol, 62.5 mM Tris–HCl, pH 6.8) before 10 min incubation at 95 °C. Proteins were separated by SDS-polyacrylamide gel electrophoresis and were transferred to PDVF membrane. Western Blot was performed using cTnI antibody (Abcam). Secondary antibody conjugated with horseradish peroxidase (Santa Cruz Biotechnology) was used for Enhanced Chemiluminescence reaction. Ponceau stain (0.1 % w/v Ponceau S, 5 % acetic acid) was used to visualize non-specific protein bands as loading control. cTnI band intensity was quantified using ImageJ software (NIH) and normalized to corresponding Ponceau loading control band intensity.

### Statistical Analysis

Student’s *t*-test was used to compare two means with normal distributions as determined by the Shapiro-Wilk test. For non-normal distributions the Mann–Whitney test was used to compare means. A *p*-value of <0.05 was used as the cutoff for statistical significance.

## Results

### DDFPe Reduces Infarct Size

**Fig. 1:**
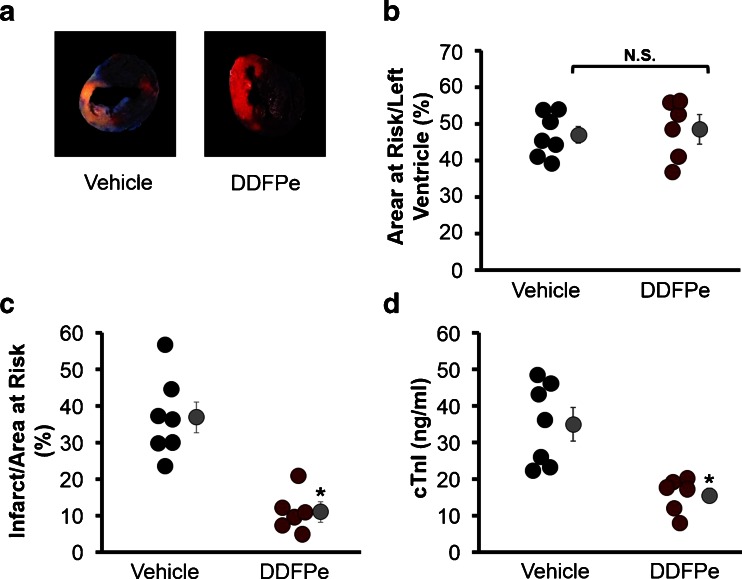
DDFPe protects the heart from myocardial infarction. Mice were administered saline vehicle or DDFPe immediately following permanent LAD occlusion. Hearts were perfused with trypan blue 24 h after LAD ligation and harvested for transverse sectioning and TTC staining (**a**) for assessment of the area at risk (**b**) and infarct size (**c**). Prior to trypan blue perfusion, heparin treated blood was collected for plasma preparation and cTnI ELISA analysis (**d**). *n* = 6–8. Means ± SEM are indicated next to each group in *grey*. * indicates significant difference (*p* < 0.05) between means, while *N.S.* indicates the means are not significantly different as determined by Student’s *t*-test

The effect of DDFPe on infarct size was quantified from TTC stained transverse cross sections of hearts from mice undergoing LAD occlusion (representative section image Fig. 1a). While vehicle and DDFPe treatment groups experienced similar sized areas at risk (46.3 ± 2.3 % for vehicle vs 47.9 ± 4.0 % for DDFPe; *p* = 0.67) (Fig. 1b), mice treated with DDFPe immediately following LAD ligation demonstrated a reduction of infarct size, decreasing from 36.9 % of the area at risk in vehicle treated mice to 10.4 % in mice receiving DDFPe (*p* < 0.01) (Fig. 1c). Release of troponin I (cTnI), a cardiac specific isoform, into the blood is a widely used marker of MI. Blood plasma ELISA assay for cTnI confirmed a reduction in the extent of cardiac injury in the DDFPe treated mice as plasma levels of cTnI were reduced from 35 ng/ml in vehicle treated mice to 16 ng/ml in the DDFPe group (*p* < 0.01) (Fig. 1d).

### Mitochondrial K_ATP_ Antagonist Prevents Infarct Reduction by DDFPe

**Fig. 2:**
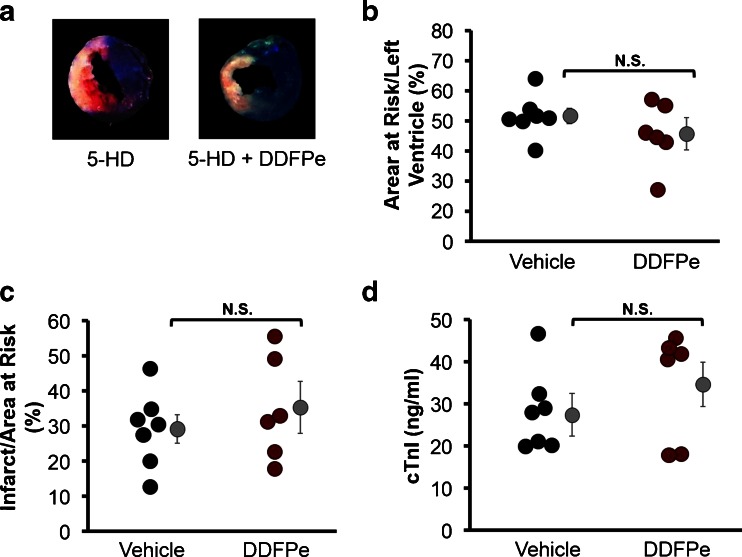
5-HD attenuates DDFPe induced cardiac protection. Mice were pretreated with 5-HD prior to LAD ligation and saline vehicle or DDFPe administration. Hearts were perfused with trypan blue 24 h after LAD ligation and harvested for transverse sectioning and TTC staining (**a**) for assessment of the area at risk (**b**) and infarct size (**c**). Prior to trypan blue perfusion, heparin treated blood was collected for plasma preparation and cTnI ELISA analysis (**d**). *n* = 6–8. Means ± SEM are indicated next to each group in *grey. N.S.* indicates that means were compared using Student’s *t*-test Mann–Whitney rank sum were not significantly different

**Fig. 3:**
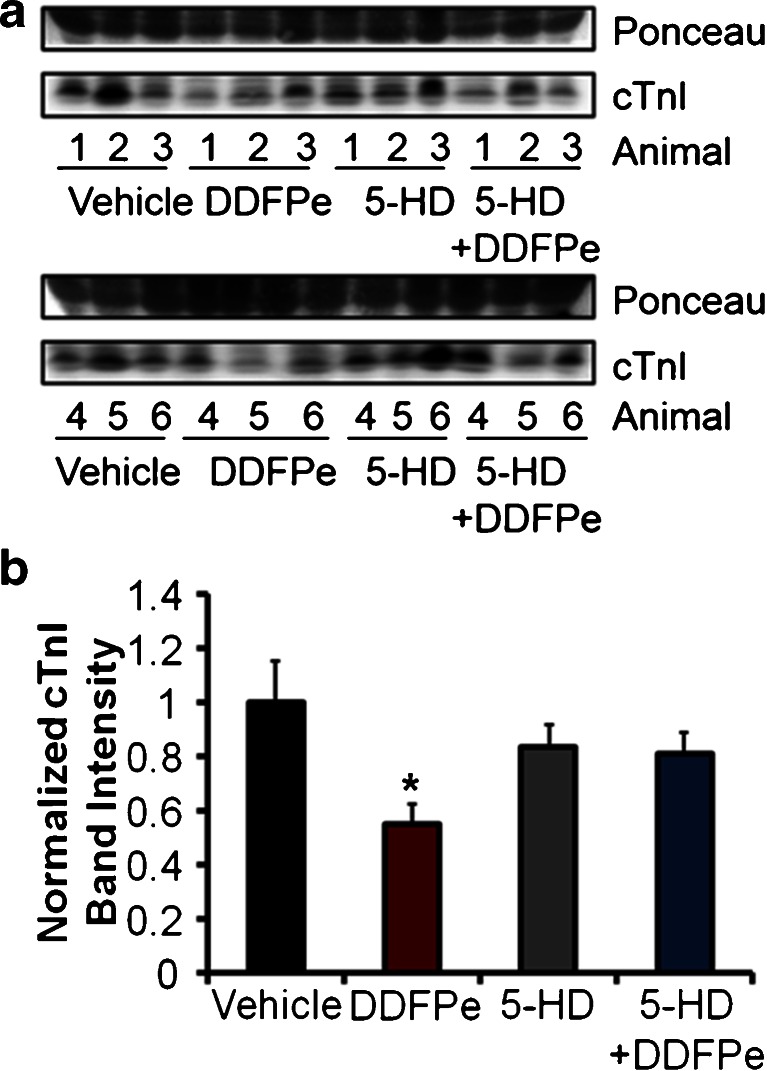
5-HD prevents the decrease in plasma cTnI levels by DDFPe. Mice were pretreated with saline vehicle or 5-HD prior to LAD ligation and saline vehicle or DDFPe administration. Blood plasma was collected 24 h after LAD ligation. Plasma samples from 6 mice in each group, indicated by animal number, were analyzed by Western blot for cTnI levels or ponceau stain for protein loading (**a**). Normalized cTnI band intensities were graphed (**b**). *n* = 6. Data is plotted as means ± SEM. * indicates significant difference (*p* < 0.05) between means as determined by Whitney-Man rank sum tests

Ischemic preconditioning is a well-established, classical method of eliciting cardiac protection from ischemic insults. Investigations into the mechanisms of cardiac protection have identified mitochondrial ATP-sensitive potassium channels (K_ATP_) as important mediators of both ischemic and pharmacological preconditioning in the myocardium. In order to investigate a potential role of DDFPe in eliciting cardiac protection through a preconditioning mechanism involving K_ATP_, mice were administered the selective mitochondrial K_ATP_ antagonist 5-hydroxydecanoate (5-HD) prior to LAD ligation. Mice administered 5-HD with or without DDFPe experienced similar areas at risk (51 % ± 2.6 for 5-HD + Vehicle vs 46 % ± 5.4 for 5-HD + DDFPe; *p* = 0.64) consistent with those for groups receiving vehicle or DDFPe alone (Fig. 2b). In mice treated with 5-HD, DDFPe failed to reduce infarct size measured by TTC staining (29.2 % ± 4.1 for 5-HD + Vehicle vs 35.4 % ± 7.4 for 5-HD + DDFPe; *p* = 0.48) (Fig. 2c). Likewise, 5-HD attenuated DDFPe associated decreases in cTnI release into the blood as measured by ELISA assay (27.4 ± 5.1 ng/ml for 5-HD vs 34.6 ± 5.3 ng/ml for 5-HD + DDFPe; *p* = 0.86) (Fig. 2d). Western blot analysis of cTnI levels in the plasma confirmed that while DDFPe alone reduced the release of cTnI into the blood following MI, this effect was blocked by pretreatment with 5-HD (Fig. 3a–b).

## Discussion

In patients experiencing an MI, the primary concern is reperfusion of the ischemic tissue through restoration of blood flow through the occluded artery. Patency of the occluded artery is often achieved clinically through the use of percutaneous coronary intervention or the use of thrombolytic agents. The time frame of reperfusion initiation is critical, and any delay can cause an increase in mortality rates [11, 12]. It is therefore imperative that re-oxygenation of the ischemic tissue occur as early as possible. PFCs have been proposed as intravenous oxygen delivery systems due to several favorable properties including oxygen dissolving and diffusion capacities, small size, inert nature, and non-extravasation [5, 6]. DDFPe was originally developed as an ultrasound contrast agent and single administration is safe in humans [13, 14]. Furthermore, DDFPe has been demonstrated to show therapeutic potential through oxygen delivery in animal models of severe anemia, hemorrhagic shock, and ischemic stroke [9, 10, 8]. The data generated here provide clear evidence for a cardiac protective role of DDFPe in the ischemic heart.

Consistent with previous studies investigating the protective effects of DDFPe in reducing infarct size in a rabbit model of stroke by permanent occlusion of the middle cerebral artery and/or anterior cerebral artery, DDFPe reduced infarct size in our mouse model of permanent LAD occlusion-induced MI. Despite experiencing a similar ischemic area, as assessed by the area at risk, mice treated with DDFPe immediately following initiation of the ischemic event by LAD ligation experienced a significantly smaller infarct. Of particular note, is the difference in DDFPe dosing regimens between our study and the work performed by Culp et al. in the rabbit stroke model. Culp et al. used repeated DDFPe injections (0.6 ml/kg) at 90 min intervals throughout the course of the experiments. In a follow up this work, it was demonstrated that DDFPe doses as small as 0.1 ml/kg given every 90 min were similarly effective in reducing infarct size in the rabbit stroke model [10]. In this study we used a single injection of DDFPe (0.6 ml/kg) administered at the onset of ischemia. These differences in dosing schedules provide valuable insight into a potential mechanism of DDFPe elicited protection against ischemia.

In order to understand the significance of the difference in dosing regimens as it relates to the mechanism of protection against ischemia, it is necessary to understand the pharmacokinetic profile of DDFPe. In normal healthy volunteers, the pharmacokinetics of DDFPe administered intravenously fits a two-compartment model. What is taken up by the various tissues presumably depends on the degree of local tissue oxygenation, however DDFPe is undetectable after approximately 8 h (personal communication with NuvOx). DDFPe is highly inert and does not undergo any detectable metabolism or biotransformation within the body. DDFPe is eliminated almost entirely through expiration and has a very short half-life of 2.2 ± 1.2 min in the blood, with nearly all DDFPe being recovered in expired air by 2 h after administration [15]. Although these studies were performed in humans, the inert nature and physiochemical properties of DDFPe suggest that a similar pharmacokinetic profile should be observable across species. Pharmacokinetic analysis of a single dose of DDFPe in rabbits revealed a comparable profile with a blood half-life of 1.45 ± 0.17 min [10].

Given the short half-life of DDFPe, the repeated 90 min dosing may be practical in order to ensure sustained oxygen delivery to the ischemic area by DDFPe. However, we were able to observe a cardiac protective effect using only a single IV dose of DDFPe in our model of 24 h LAD occlusion. As DDFPe should be eliminated from the system well before 24 h, it raises the question of how a single dose of DDFPe is able to reduce infarct size so dramatically. Similarly, in a follow-up to the work done by Culp et al., it was observed that even with repeated dosing, DDFPe maintained neuroprotection beyond its retention time in the body [10]. We propose that oxygen delivery by DDFPe to the ischemic tissue is slowly diminished over time as DDFPe is eliminated, resulting in an increasingly hypoxic environment. We believe this slow advancement to hypoxia is able to reduce infarct size by mimicking certain events that occur during ischemic preconditioning (Fig. 3).

**Fig. 4:**
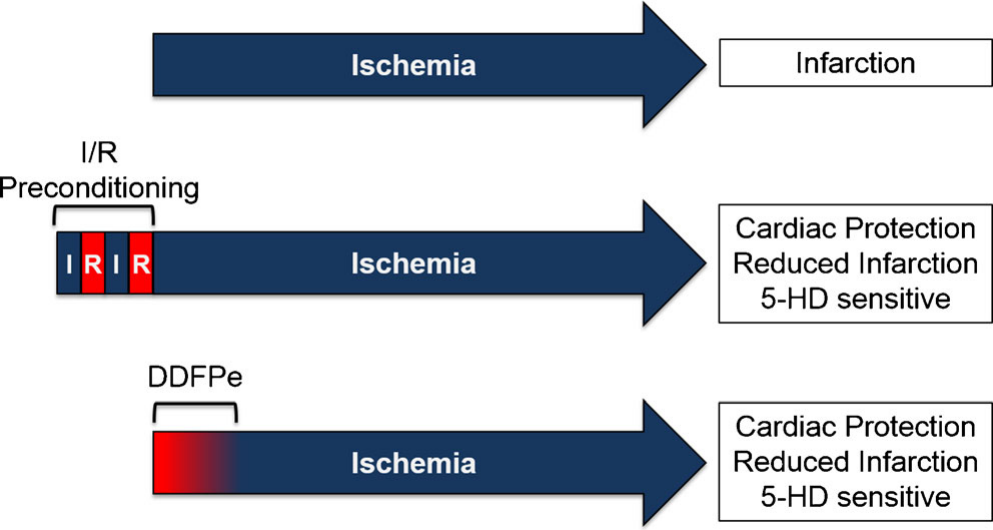
Proposed Model of Cardiac Protection by DDFPe. *Blue* indicates periods of ischemia (I), while *red* represents periods of reperfusion (R) or oxygen delivery by DDFPe. The *red* to *blue* color fade-in depicted during DDFPe period represents the proposed gradual decrease in oxygen delivery that occurs as DDFPe is eliminated from the system

Ischemic preconditioning (IPC) is a well characterized phenomenon, whereby short cycles of ischemia and reperfusion are able to protect the heart from subsequent long term ischemic insults. IPC initiates a plethora of signaling events mediated by triggers such as adenosine, bradykinin, and opioids [16]. These IPC triggers converge on several downstream mediators to elicit the protective effects. A key mediator of IPC is activation of the mitochondrial ATP-sensitive potassium channel (mitoK_ATP_) [17]. While direct activation of mitoK_ATP_ by diazoxide is sufficient to elicit pharmacological IPC, the mitoK_ATP_ inhibitors glibenclamide and 5-hydroxydecanoate (5-HD) block both classical IPC and as well as many methods of pharmacological IPC, highlighting its importance [18–21].

In order to elucidate a potential role of mitoK_ATP_ and IPC in DDFPe-induced cardiac protection, the mitoK_ATP_ inhibitor 5-HD was utilized. 5-HD was chosen over glibenclamide based on greater specificity for the mitoK_ATP_ over the sarcoplasmic K_ATP_ channel [22–24]. Pretreatment with 5-HD prior to LAD occlusion did not appreciably alter infarct size (36.9 ± 4.2 % for vehicle vs 29.2 ± 4.4 % for 5-HD) or cTnI release (35.0 ± 4.6 ng/ml for vehicle vs 27.4 ± 5.1 ng/ml for 5-HD). However, 5-HD pretreatment blocked the decrease in infarct size associated with DDFPe alone, supporting an IPC-like event mediated by mitoK_ATP_ as the mechanism of DDFPe cardiac protection, as illustrated in Figure 4.

Our results demonstrated that DDFPe elicits a cardiac protective effect that persists well beyond the assumed elimination time of DDFPe. The initiating events that lead to sustained cardiac protection are likely the result of the oxygen delivery provided by DDFPe early on, as DDFPe is highly inert and has not been reported to interact directly with any signaling pathways. However, our data indicate that DDFPe elicits protection through the same mediator as many IPC triggers, providing potential leads to investigate the exact signaling pathways responsible. Perhaps the most likely trigger is an increase in reactive oxygen species (ROS), which have been identified as an important mediator required for both mitoK_ATP_ activation and IPC [25–27]. The increased generation of ROS has been a potential concern with enhanced oxygen delivery systems such as DDFPe, but the influence of DDFPe on ROS production in vivo has yet to be investigated. Although, the deleterious effects of ROS in numerous pathologies have been well documented, ROS can also play a protective role in low dose or short-term exposures, as with IPC. If DDFPe does in fact increase ROS production, it is possible that a single administration of DDFPe may be more advantageous to elicit a preconditioning effect without the subsequent damage caused by excessive ROS from repeated dosing.

One of the pitfalls of IPC is the necessity for the preconditioning to occur prior to ischemia, which is impractical in the clinical setting as patients presenting to the emergency room have already experienced an ischemic event. However, it has been observed that many of the initiating triggers of IPC also elicit protection when initiated prior to reperfusion, a process termed post-conditioning [28]. In addition to being initiated by many of the same triggers as IPC, post-conditioning is mediated by similar mechanisms, including activation of the mitoK_ATP_ [28, 29]. The dependence of DDFPe on mitoK_ATP_ for cardiac protection in this model suggests that DDFPe may be effective in initiating post-conditioning like protection as well. Although this study did not address the utility of DDFPe on post-conditioning, studies investigating the protective potential of DDFPe administered after a period of ischemia, but prior to complete reperfusion are warranted. It is particularly important to assess the protective potential of DDFPe in a model of reperfusion as this is more clinically relevant to revascularization that occurs following an acute myocardial infarction.

This observation that DDFPe is able to stimulate cardiac protection through an IPC-like mechanism represents an exciting discovery in the field of PFC oxygen delivery. The sole therapeutic potential of PFC has always been assumed to stem from the gas delivery properties, specifically the ability to supplement oxygen delivery; however, the data presented here suggests that the protective effects extend beyond the timeframe of elimination from the system. Such a lasting protective effect offers many potential benefits that may be applied clinically. As the time to reperfusion initiation is critical to limiting myocardial damage following an ischemic event, the use of DDFPe can extend the window of optimal reperfusion. Myocardial cell injury can occur within 20–30 min of ischemia; however, myocardial necrosis can take several hours to develop and even a 1–2 h extension in the timeframe of optimal reperfusion would have significant impact on survival and recovery from MI. In addition, even in the absence of reperfusion DDFPe produced dramatic cardiac protection observable out to 24 h after LAD occlusion. As reperfusion is the most important therapy to reduce myocardial injury following an ischemic, DDFPe may have a more pronounced protective effect when combined with reperfusion.

DDFPe has already been demonstrated to be safe in humans. Moreover, DDFPe is stable when stored at room temperature which makes it an ideal candidate for future development into clinical practice as it may be administered in the initial treatment of ischemic events by first responders. Furthermore, if DDFPe is in fact initiating mechanisms of IPC it may offer additional therapeutic potential. The protective effects of IPC are not limited to the myocardium and IPC has been shown to be protective in other ischemic tissue as well, including stroke and organ transplantation, suggesting a potential use of DDFPe IPC-like protection in these applications [30–33].
